# Heterovalent Substitution to Enrich Electrical Conductivity in Cu_2_CdSn_1-x_Ga*_x_*Se_4_ Series for High Thermoelectric Performances

**DOI:** 10.1038/srep09365

**Published:** 2015-03-20

**Authors:** Bo Wang, Yu Li, Jiaxin Zheng, Ming Xu, Fusheng Liu, Weiqing Ao, Junqing Li, Feng Pan

**Affiliations:** 1 College of Materials Science and Engineering, Shenzhen University and Shenzhen Key Laboratory of Special Functional Materials, Shenzhen 518060, People's Republic of China; 2 School of Advanced Materials, Peking University, Shenzhen Graduate School, Shenzhen 518055, People's Republic of China

## Abstract

Serials of Ga doping on Sn sites as heterovalent substitution in Cu_2_CdSnSe_4_ are prepared by the melting method and the spark plasma sintering (SPS) technique to form Cu_2_CdSn_1-*x*_Ga*_x_*Se_4_ (*x* = 0, 0.025, 0.05, 0.075, 0.01, and 0.125). Massive atomic vacancies are found at *x* = 0.10 by the heterovalent substitution, which contributes significantly to the increase of electrical conductivity and the decrease of lattice thermal conductivity. The electrical conductivity is increased by about ten times at 300 K after Ga doping. Moreover, the seebeck coefficient only decreases slightly from 310 to 226 μV/K at 723 K, and a significant increase of the power factor is obtained. As a result, a maxium value of 0.27 for the figure of merit (*ZT*) is obtained at *x* = 0.10 and at 723 K. Through an *ab initio* study of the Ga doping effect, we find that the Fermi level of Cu_2_CdSnSe_4_ is shifted downward to the valence band, thus improving the hole concentration and enhancing the electrical conductivity at low doping levels. Our experimental and theoretical studies show that a moderate Ga doping on Sn sites is an effective method to improve the thermoelectric performance of Cu_2_CdSnSe_4_.

Based on the Seebeck effect or the Peltier effect, thermoelectric (TE) materials can convert heat to electricity directly and vice versa. Therefore they have a great potential to be used as power generators by recycling waste heat or coolers by using electrical energy. The efficiency of a TE material is determined by the dimensionless figure of merit: *ZT* = *S*^2^*σT*/*κ*, where *S*, *σ*, *κ*, and *T* are the Seebeck coefficient, electrical conductivity, total thermal conductivity, and absolute temperature, respectively. Thermal conductivity is mainly composed of carrier thermal conductivity (*κ_c_*) and lattice thermal conductivity (*κ_l_*). *S*^2^*σ* is defined as Power Factor (*PF*) to evaluate the electric properties of a TE material. In order to obtain good TE performance, a relatively high *S* and *σ* and low *κ* are needed. Because these parameters are strongly interdependent and confined to the TE material types, it is an intensively studied and challenging topic to optimize such parameters.

Cu_2_-(Cd/Zn)-(Sn/Ge)-Se_4_ type quaternary compounds such as Cu_2_CdSnSe_4_ and Cu_2_ZnSnSe_4_, which are associated with intrinsically low thermal conductivities, are reported as potential *p*-type TE materials in middle temperature^1^,^2^,^3^,^4^,^5^. The band gaps are 0.89 eV for Cu_2_CdSnSe_4_ and 1.41 eV for Cu_2_ZnSnSe_4_^4^, which are much larger than those of traditional thermoelectric materials. The wide band gap property of such quaternary compounds helps enhance their TE performances, because the narrow band gap in traditional TE materials introduces a bipolar effect to reduce the thermoelectric efficiency. The reported *ZT* values for pure polycrystalline are 0.19 for Cu_2_CdSnSe_4_ and 0.18 for Cu_2_ZnSnSe_4_^4^. Many efforts have been done to enhance the TE performance of these quaternary compounds by doping^1^,^4^,^6^,^7^,^8^,^9^,^10^,^11^ and nanocrystalling^3^,^5^,^12^,^13^,^14^,^15^,^16^,^17^,^18^. Nanocrystallization is an effective way to reduce the thermal conductivity, but the complex chemical procedure is not suitable for large scale production in commercial applications. Doping by atomic substitutions has been mainly reported of Cd/Zn sites replaced by Cu^3^,^4^,^19^,^20^, Mn^21^ or Fe^8^,^10^, Sn sites by In 7,22, and Se sites by S^6^,^9^,^11^ to enhance the electrical performance. Among these efforts, Cu-doping at Cd/Zn site is the most effective way till now. Not only is the electrical conductivity enhanced, but also the thermal conductivity is reduced^4^,^15^.

Heterovalent substitution is an effective approach to directly affect the Fermi level and further increase the carrier concentration. For example, doping Ga atoms in Cu_2_Ga_0.075_Sn_0.925_Se_3_^23^ increased the electrical conductivity from 12600 to 726000 Sm^−1^ at room temperature. Just like Cu_2_SnSe_3_^24^, Cu_2_CdSnSe_4_ also shows a diamond-like crystal structure, Cu, Cd, and Sn atoms bonded with Se atoms to form tetrahedral coordination. Using *ab initio* calculations, it is found that the electrical conductivities of these Cu-based ternary or quaternary diamond-like compounds are mainly generated from the hybridization of Cu 3*d* and Se 4*p* orbitals near the valence-band maximum (VBM)^25^. Though Sn orbitals contribute little to the hole concentration, they offer electrons to valence bands close to the VBM. In principle, the Sn sites should be a good choice to optimize properties in Cu-based diamond-like compounds. If the conductive Cu-Se bond network was considered as the 3D framework of the compound, the behavior of the differently bonded Sn atoms is pretty much like the filler atoms in filled skutterudites. Thus, the great potential emerges for the Sn sites doping to enhance phonon scattering or electrical conductivity inside the diamond-like structures^24^.

In this work, we provide the first report on the effect of the heterovalent substitution of Ga doping at Sn site of the crystal structure and the TE performance for Cu_2_CdSn_1-*x*_Ga*_x_*Se_4_. A significant increase of electrical conductivity and power factor has been observed, and the *ZT* value has been improved from 0.19 to 0.27 at 723 K. To explore the mechanisms behind the experimental results, we have performed an *ab initio* study on the Ga doping effect and find that the Fermi level of Cu_2_CdSnSe_4_ is shifted downward to the valence band, thus improving the hole concentration and enhancing the electrical conductivity at the low level doping content.

## Results and discussion

### Crystal structure and phase compositions

**Figure 1:**
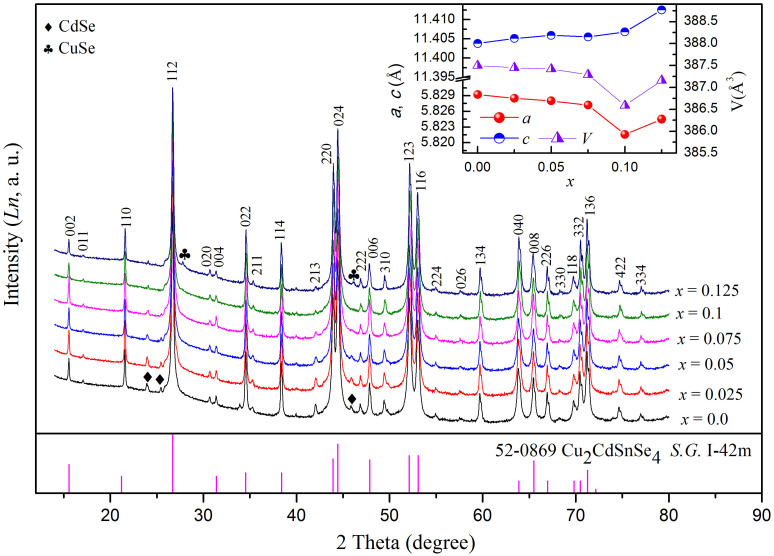
The XRD patterns of serials of Cu_2_CdSn_1–x_Ga_x_Se_4_ samples after SPS. The inset shows the lattice parameters *a*, *c*, and *V* of Cu_2_CdSn_1–x_Ga_x_Se_4_ solid solutions a function of the Ga content *x*.

XRD-patterns of the SPS sintered samples are shown in Fig. 1. The diffraction intensities are shown in the natural logarithm (Ln) manner in order to distinguish the weak peaks. It reveals that all of the samples are stannite-type structure Cu_2_CdSnSe_4_ with a trace amount of CdSe or CuSe phases. The impurity phase is mainly CdSe phase for *x* ≤ 0.075, and CuSe phase for *x* = 0.10 and 0.125. The two weak peaks between 2θ = 38.9° and 2θ = 40.5° are the residual *K_β_* peak of (220) and (024) diffraction peaks. The residual *K_β_* peak of the strongest diffraction peak (112) is located at about 2θ = 24.08°, which is overlapped with the (100) peak of CdSe at 2θ = 23.90° of hexagonal CdSe to form a broad peak. The sharper peak at this position in the XRD patterns of *x* = 0.10 and 0.125 samples indicated nonexistence of CdSe phase in the two samples. Chemical composition analysis of the phase by electron probe microanalysis (EPMA) confirms the XRD results. A white or deep gray unregular regions in EPMA image also indicate the existence of CdSe or CuSe (Fig. S1). The compositions of Cu_2_CdSn_1-*x*_Ga*_x_*Se_4_ phases analyzed by EPMA are listed in Table 1. The normalized chemical formula are based on fixing the number of Cu atom to 2, because Cu has the lowest vapor pressure in this system, thus the loss will be the lowest among these element during the procedure of sample preparation. The results indicate that all of the samples are deficient of Se, and the content of Ga is lower than the nominal composition. Vapor lost may be the main reason because of the high vapor pressure of Ga and Se. But the increasing or decreasing tendency of the Ga or Sn component agrees with the nominal composition.

**Figure 2:**
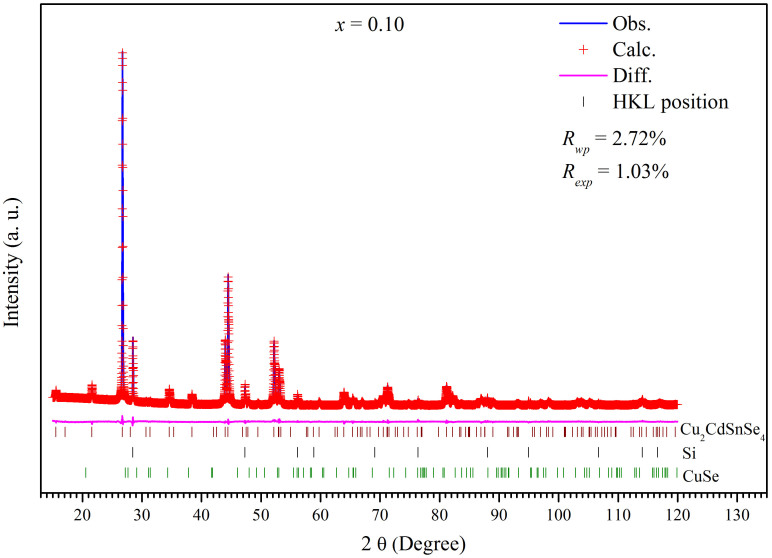
Rietvold refinement for the XRD pattern of the samples *x* = 0.10.

The Rietveld refinement was performed on the XRD Patterns of the samples with a 2θ range of 10°–120° by using an internal Si standard as calibration. The Cu_2_CdSnSe_4_ shows a tetragonal stannite-type 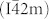 structure. The Cu, Cd, and Sn atoms occupy at the special Wyckoff positions 4d (0, 0.5, 0.25), 2a (0, 0, 0), and 2b (0, 0, 0.25), respectively, and the Se atom occupies at the 8i (0.255, 0.255, 0.137) position. The lattice parameters, reliable factor*s*, and the weight percent of the impurity phase CdSe or CuSe are shown in Table 2. The atomic bond-length between Se and Cu, Cd or Sn atoms and the fractional coordinates are shown in Tab. S1. The Rietveld refinement shows that the Ga doping slightly changes the atomic position of Se. Due to the array detector was used, the strong intensity of the pattern results in slightly large Gof factor, but the low *R_wp_* and *R_Bragg_* factors (Table 2) indicate the good reliability of the Rietveld refinement results. A represent Rietveld refinement of XRD pattern for the sample of *x* = 0.10 is shown in Fig. 2. When all the atomic positions of Cu, Cd, and Sn are opened for Ga atom during the Rietveld refinement, Ga atoms prefer to take the Sn position. It may be due to the more close cationic radius between Ga^3+^ (0.47 Ả) and Sn^4+^ (0.55 Ả)^26^ than others.

The inset of Fig. 1 shows the variation of lattice parameters of *a*, *c*, and *V* as the Ga content *x*. The values of *a*, *c*, and *c/a* for the undoped sample in our work are almost the same as the reported data^9^,^27^. The *a* axis and cell volume first decrease slightly with the increasing Ga content from *x* = 0 to *x* = 0.075, indicating that the Ga atoms do incorporate in the crystal structure of Cu_2_CdSnSe_4_. While the *c* axis slightly increases with the increasing *x*, it may account for the bonding anisotropy in the *ab* plane and along the *c* direction. The lattice parameter *a* and cell volume decrease dramatically to a valley bottom when the Ga doping content is increased from *x* = 0.075 to *x* = 0.10. This is because under low content of trivalent Ga^3+^ cation substitution at the tetravalent Sn^4+^ cation sites, the lattice distortion would compensate the unbalance of charge caused by heterovalent substitution. If the content of substitution is further increased, lattice vacancies of Se will be produced, thus leading to a further decrease of the lattice parameter *a* and cell volume. The compositions of Cu_2_CdSn_1-*x*_Ga*_x_*Se_4_ phases analyzed by EPMA (Table 1) indicate that the Se molar percentage in the sample of *x* = 0.10 is lower than others. Finally, due to more Cu atoms getting to form the CuSe phase in the sample of *x* = 0.125, the relative excess Sn or Cd atoms may occupy the Cu sites to balance the charge caused by the substitution Ga^3+^ of Sn^4+^. Therefore, the content of Se vacancies gets lower, which leads to the increase of lattice parameter.

### Electric transport properties

**Figure 3:**
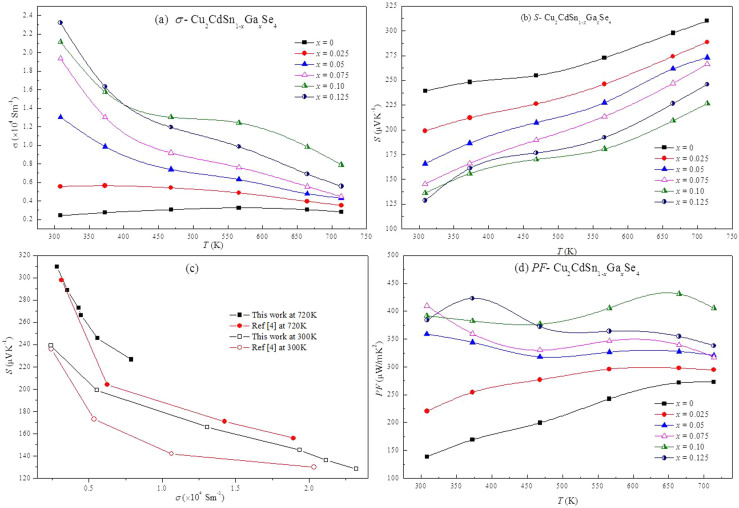
Electrical transport properties of Cu_2_CdSn_1-x_Ga_x_Se_4_ samples as a function of temperature. (a) electrical conductivity(*σ*); (b) seebeck coefficient (*S*); (c) the relationship between electrical conductivity and seebeck coefficient in this work and other reported system; (d) power factor (*PF*).

Fig. 3a shows the temperature dependence of electrical conductivity of Cu_2_CdSn_1-x_Ga*_x_*Se_4_. The electrical conductivity of the undoped sample (2426 Sm^−1^) is the same as reported by Liu *et al*.^4^ at room temperature. Interestingly, the electrical conductivity of the undoped sample increases a little bit with the increasing temperature, but for Ga-doped samples, it decreases with the increasing temperature and exhibits a degenerate semiconducting behavior. The reason of such phenomenon will be explained in the calculation part. As the increase of the doping content *x*, the incorporation of Gallium gradually improved the value of *σ* of Cu_2_CdSn_1-x_Ga*_x_*Se_4_ at the whole temperature range except the sample of *x* = 0.10. The value of *σ* for the sample *x* = 0.10 is higher than that of *x* = 0.125 when the temperature is above 450 K. It is about ten times magnifying power as the doping content increases from 0 to 0.125 at room temperature. Hall measurements indicate that the increase of *σ* is mainly due to the gradual increase of carrier concentration and carrier mobility by doping (Table 2). The carrier concentration increases dramatically as expected, which is attributed to that the substitution of quadrivalent Sn^4+^ by trivalent Ga^3+^ introduces more hole carriers. The Hall measured mobility increases from 14.14 to 53.9 cm^2^V^−1^s^−1^ with *x* increased from *x* = 0 to *x* = 0.075 then declines to 19.2 cm^2^V^−1^s^−1^ for *x* = 0.10, and finally increases again to 21.6 cm^2^V^−1^s^−1^ for *x* = 0.125. It hard to clarify the mechanism for the variation of carrier mobility with the increasing Ga doping content from *x* = 0 to *x* = 0.075. However, the difference in Hall measured carrier mobility is also very small, which is in the range of measurement errors.

Seebeck coefficients of Cu_2_CdSn_1-x_Ga*_x_*Se_4_ as a function of the temperature are shown in Fig. 3b. The Seebeck coefficients show positive values and increase with the temperature nearly linearly for all samples over the temperature range between 300 K and 723 K. Note that all Seebeck coefficients of Ga-doped samples are lower than the undoped one at the temperature range of 300 K to 723 K, and the variation tendency of Seebeck coefficients is just the opposite to the variation of electrical conductivity for the Ga-doped samples. The reason is that, in most cases, the lower resistivity (higher electrical conductivity), the lower Seebeck coefficient is, because both of Seebeck coefficient and resistivity are related to the inverse proportion with the carrier concentration and mobility. Compared with the electrical transport data of Cu-doping on Cd at room temperature^4^, it can be found that the *σ* of Cu_2_CdSnSe_4_ is improved more effectively by Ga-doping on Sn site, but drops faster with the increasing temperature than Cu-doping, which is not beneficial for the thermoelectric performance of the material at high temperature. However, Ga-doping holds higher value of Seebeck coefficient than Cu-doping at the same electrical conductivity level, as shown in Fig. 3c. The probable reason will be discussed in the calculation part.

Under a given temperature difference, the ability to produce useful electrical power for a material is quantified by its power factor (*PF*): *PF* = *S*^2^/*ρ*. As shown in Fig. 3d, Ga-doping improves the *PF* from 273 μW/m/K^2^ for the undoped sample to 405 μW/m/K^2^ for the samples of *x* = 0.10 at 723 K, nearly by a factor of 1.5. The power factor of 405 μW/m/K^2^ obtained here is comparable to that of the case of Cu-doping on Cd sites at 700 K (about 450 μW/m/K^2^)^4^ and is moderate compared of other state-of-the-art thermoelectrics^28^,^29^,^30^. The reason for the improved power factor is mainly attributed to the increased electrical conductivity by Ga-doping. There is an irregular *T* dependence in electrical transport properties (oscillating trend) for the simples with high Ga-doping content, which mainly occurs below 400 K. We attribute this to the phase transformation for the impurity CuSe phase at 51°C (324 K) and 120°C (393 K) (Fig. S2), which would lead to the variation of the electrical transport properties.

### Thermal transport properties

**Figure 4:**
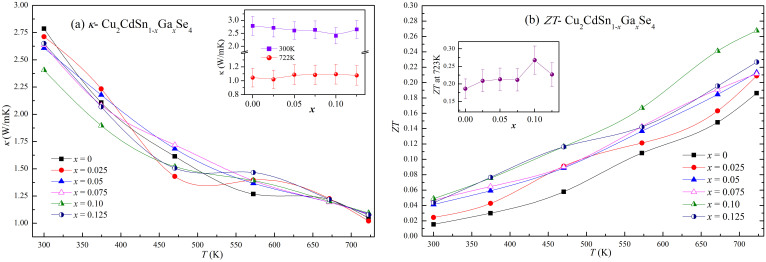
Temperature dependence of thermal transport properties of Cu_2_CdSn_1–x_Ga_x_Se_4_ samples and *ZT* value. (a) Total thermal conductivity (*κ*), the inset shows the relationship between *κ* and the Ga content *x* at 300 K and 723 K, respectively; (b) Dimensionless figure of merit (*ZT*) of Cu_2_CdSn_1–x_Ga_x_Se_4_ as a function of temperature. The inset shows the ZT value as a function of the Ga content *x*.

The temperature dependent of the total thermal conductivity for all the samples are displayed in Fig. 4a. The total thermal conductivity of the undoped sample is 2.9 Wm^−1^K^−1^ at room temperature, nearly the same as that obtained by Min-Ling Liu *et al.* for the undoped samples^4^. The carrier contribution (*κ_c_*) was calculated from the electrical conductivity by using the Wiedemann-Franz relation, *κ_c_* = *LTσ*, with a Lorentz number *L* = 1.50 × 10^−8^ W/Ω/K^2^ (Fig. S3a). The remaining lattice contribution (*κ_l_* = *κ* - *κ_c_*) is plotted in Fig. S3b, and we can see that values of *κ_l_* are more than one order larger than that of *κ_c_*, indicating that the total thermal conductivity mainly comes from the lattice phonon contribution. The inset of Fig. S3b shows the Ga content dependence of *κ_l_* of the samples at 300 K and 723 K. We can see that at 300 K, the *κ_l_* decreases gradually with the increase of Ga content except for the drastic decrease for the sample of *x* = 0.10. The substitution of Sn by Ga raises the lattice distortion and thus enhances the phonon scattering, which accounts for the first gradual decrease for the *κ_l_*. For the sample of *x* = 0.10, the atomic vacancies produced by the massive non-equivalent substitution may contribute to the drastic decrease of *κ_l_*.

Fig. 4b shows the temperature dependence of the *ZT* value. Obviously, *ZT* values increase with the temperature monotonously due to the decrease of *κ*. The *ZT* value of the un-doped sample is 0.19, which is almost the same as the reported value for Cu_2_CdSnSe_4_^4^. The *ZT* value increases with the increasing Ga-doping content *x* up to 0.10, then slightly decreases at *x* = 0.125 (Fig. 4b Inset), due to the Ga doping content dependence of the *PF* and the unchanged *κ_l_* with the increasing Ga doping content at 723 K. A maximum *ZT* value of 0.27 is obtained at *x* = 0.10, which is about 1.4 times higher than the un-doped sample. This value is lower than the reported *ZT* = 0.65 for Cu-doped Cu_2.10_Cd_0.9_SnSe_4_ at 700 K^4^, which is mainly attributed to that the Cu-doping dramatically suppressed the thermal conductivity to 0.49 at 700 K. If we combine the high Seebeck coefficient of Ga-doping on Sn and the low thermal conductivity of Cu-doping on Cd, the thermoelectric performance would be improved to a new high level. So Ga and Cu co-doping on Cu_2_CdSnSe_4_ may be a good way to further enhance the thermoelectric performance.

### Calculation results

**Figure 5:**
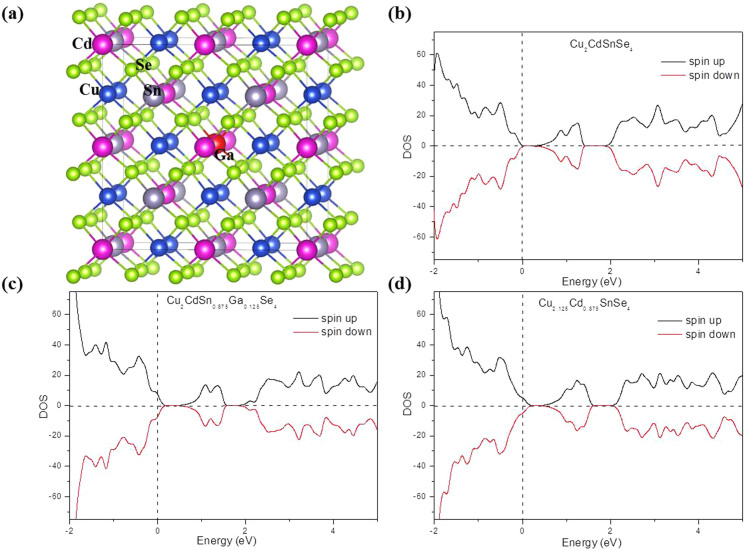
DFT calculation results. (a) Configuration of Cu_2_CdSn_1–x_Ga_x_Se_4_ for *x* = 0.125. For Cu_2_CdSn_0.875_Ga_0.125_Se_4_, the doped-Ga and its adjacent atoms are highlighted in ball and stick model to give an overall picture of the doping structure. (b–d) Total density of states (DOS) for Cu_2_CdSnGaSe_4_ (b), Cu_2_CdSn_0.875_Ga_0.125_Se_4_ (c), and Cu_2.125_Cd_0.875_SnSe_4_ (d). The Fermi energy of each model is set to 0.

In order to identify the effect of Sn atoms partially substituted by Ga atoms to the thermoelectric performance of Cu_2_CdGa_x_Sn_1-x_Se_4_, we performed a density functional theory (DFT) study of the density of states (DOS) for our materials. We have considered *x* = 0, 0.125, and 0.25, and Fig. 5a shows the doping model of *x* = 0.125. Fig. 5b and 5c show the DOS for Cu_2_CdGa_x_Sn_1-x_Se_4_ with *x* = 0 and *x* = 0.125, respectively. Our calculation clearly reveals a gradual shift of the Fermi level toward the valence band with Sn substituted by Ga and the Fermi level shift reached up to 0.18 eV at *x* = 0.25 as shown in Fig. S4. The extents of the Fermi level shift coincide with the doping levels and the increasing hole concentration as observed in experimental results. By contrast, the band gap is nearly unchanged when the Sn atoms are substituted by the Ga atoms (Fig. S4). The Fermi level shift downward to the valence band maximum (VBM) shows that there is an *p*-type doping effect when Sn substituted by Ga for Cu_2_CdSnSe_4_.

**Figure 6:**
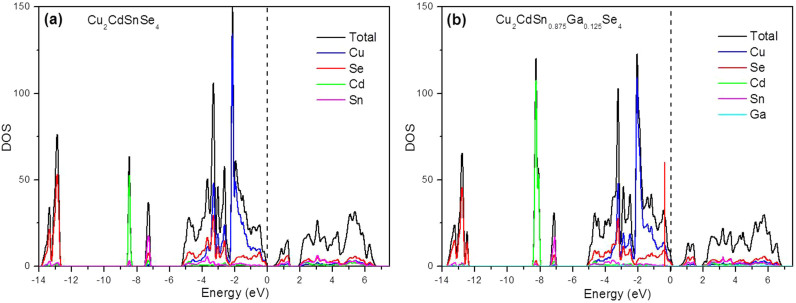
Total and projected density of states (DOS) for Cu_2_CdSn_1–x_Ga_x_Se_4_ when *x* = 0 (a) and 0.125 (b).

In Fig. 6, we show the calculated projected DOS (PDOS) of the intrinsic Cu_2_CdSnSe_4_ and Cu_2_CdGa_0.125_Sn_0.875_Se_4_, respectively. To aid the analysis of the PDOS, we present the model of Cu_2_CdGa_0.125_Sn_0.875_Se_4_ with the doped-Ga and its adjacent atoms highlighted in ball and stick mode (Fig. 5a). The PDOS near the Fermi level is mainly from the Se-Cu hybridized orbitals for both the intrinsic and doped systems. After doping with Ga atoms, the bonding of Ga-Se is relatively weaker than Sn-Se bonds, since the valence electrons of Ga is less than Sn. One main change of the PDOS after doping is that the Cu-3*d* orbitals have a second sharp peak (denoted with vertical red line) at *E*−*E*_F_ = −0.38 eV hybridized with the Se-4*p* orbitals, indicating stronger bonding between Cu and Se atoms. The enhanced Cu-Se bonding is ascribed to the doped Ga-Se bonds which are weaker than the Sn-Se bonds: as the attraction between Ga/Sn-Se gets weakened after doping, the Se has more priority to bond with Cu atoms. Moreover, the enhanced Cu-Se interaction is further validated by checking all the optimized Ga-doped structures, and the Cu-Se bonds for all doped simples are found to be shortened. For example, the Cu-Se bond of Cu_2_CdSn_0.875_Ga_0.125_Se_4_ is shortened from 2.448 Å for the pure Cu_2_CdSnSe_4_ to 2.431 Å. Such electronic variations account for the energy decrease of the Se-Cu states, and hence induce the Fermi level shift downward to the valence band, which is supposed to be the *p*-type doping effect and contributes to the degenerate semiconducting behavior of its electrical conductivity for Ga-doped samples (the *σ* decreases with the increasing temperature), as mentioned in the part of electric transport properties. The *p*-type doping effect will increase the hole concentration and improve the electrical conductivity, as suggested by our experimental results when Sn substituted by Ga at a degree of *x* = 0.1 and 0.125. The Rietveld refinement results show the Cu-Se bond is nearly unchanged after Ga-doping (Tab. S1), which may be attributed to the large amounts of Se vacancies and the phase impurities in the samples, and the error for the Rietveld refinement.

Compared with Cu-doping on Cd sites at the same doping level (Fig. 5d), the degree of Fermi level shift for Ga-doping on Sn sites is smaller under the same doping content, indicating a larger effective mass for Ga-doping. This is attributed to that both Ga-doping and Cu-doping can enhance the bond strength of Cu-Se, but the degree for Cu-doping is larger (for example, the Cu-Se bond of Cu_2_CdSn_0.875_Ga_0.125_Se_4_ is shortened from 2.448 Å for the pure Cu_2_CdSnSe_4_ to 2.431 Å. By contrast, the Cu-Se bond of Cu_2.125_Cd_0.875_SnSe_4_ is shortened from 2.448 Å for the pure Cu_2_CdSnSe_4_ to 2.428 Å). As for the degenerate semiconductors, the Seebeck coefficient can be expressed as^29^:
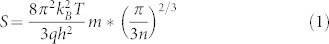


with
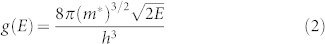


where *n* is the carrier concentration, m* is the effective mass of the carrier, and *g*(*E*) is the density of states. According to equation (2), due to the more DOS around the valence band maximum (VBM) (Fig. 5c and 5d), the Ga-doping on Sn sites introduces larger hole effective mass than the Cu-doping on Cd sites does under the same doping content. Thus, the Ga-doping would hold higher value of Seebeck coefficient than Cu-doping at the same electrical conductivity level, also as mentioned in the part of electric transport properties.

According to our calculation results, the DOS near the VBM is nearly unchanged after the Ga-doping on Sn sites, and the effective mass should keep unchanged. However, Ga contents could introduce more impurity scattering centers to impede the electron transport, thus still leading to the decreased carrier mobility. However, the Hall measurements show higher carrier mobility after the Ga-doping on Sn sites. Similar phenomenon that the carrier mobility increases after doping foreign atoms is also reported in other experimental works, such as for In dopant in Cu_2_ZnSnSe_4_^31^ and Mg dopant in Cu_2_ZnSnSe_4_^32^, and the reasons are still far from understood. More intensive and detailed work needs to be done in the future to fully clarify this. In our work, as mentioned in the part of electric transport properties, one most probable reason accounting for the above discrepancy is that the differences in the measured carrier mobility are very small, which in the range of measurement errors. Meanwhile, the impurity CdSe phase would introduce large carrier mobility (660 cm^2^V^−1^s^−1^), thus to improve the whole carrier mobility.

## Conclusions

Thermoelectric performance for serials of Cu_2_CdSn_1-*x*_Ga*_x_*Se_4_ solid solution is studied both experimentally and theoretically. It is shown that heterovalent substitution of Sn by Ga is an effective way to improve the electrical conductivity, and it also holds relatively high Seebeck coefficient. A maximum value of *ZT* = 0.27 is obtained at *x* = 0.10 and 723 K. Our Hall measurements and DFT calculation results prove that the intrinsic doping effect caused by the substitution of Sn with Ga could introduce higher hole concentration to enhance the electrical conductivity. We finally propose that Ga and Cu co-doping in Cu_2_CdSnSe_4_, such as for the high Seebeck coefficient of Ga-doping on Sn and for the low thermal conductivity of Cu-doping on Cd, may be a good way to further enhance the thermoelectric performance.

## Methods

### Experimental procedure

The stoichiometric compounds Cu_2_CdSn_1-*x*_Ga*_x_*Se_4_ (*x* = 0, 0.025, 0.05, 0.1, 0.125) are prepared by the melting and the subsequent spark plasma sintering (SPS) method. The starting materials of the elements of Cu, Cd, Ga, Sn, and Se with a purity of 99.99% mixture were sealed in the evacuated quartz tube and heated at 1237 K for homogeneity for 172 h in the muffle furnace, and then cooled down to 723 K to react for 72 h, and then subsequently quenched in the liquid nitrogen. The quenched alloys were powdered in the agate mortar and then ball-milled in a planetary ball millor (QM-4F, Nanjing University, China) by using a hard stainless steel vial and zirconia balls, at 200 rpm for 12 h. The weight ratio of ball to powder was kept at about 20:1, and the mill vial was evacuated and then filled with the purified H_2_ gas to prevent the powder from oxidation during the milling process. The milled powders were pressed as pills, and then sealed in the evacuated quartz tube to react at 723 K for 172 h again, and then the pills were ball-milled to powders. The powders were consolidated by SPS at 923 K for 5 min under an axial pressure of 48 MPa with a peak impulse value of 675 A to obtain the high density samples.

The bar specimens with a typical dimension of 12.0 mm × 5.0 mm × 5.0 mm were prepared for the electronic property measurements, and the disk specimens with 10.0 mm in diameter and 2.0 mm in thickness for the thermal conductivity measurements. X-ray powder diffraction (XRD) data were collected by a Bruker D8 Advance SS/18 kW diffractometer with the CuKα radiation. Accurate lattice parameters were got by the Rietveld refinement method with Topas 3.1 software^33^,^34^. The Seebeck coefficient (*S*) and the electrical conductivity (*σ*) were measured by using ZEM-2 (Ulvac-Riko, Japan) in the helium atmosphere. The thermal conductivity (*κ*) was calculated by using the equation *κ = λC_p_d*, where *λ* is the thermal diffusivity, *C_p_* is the heat capacity, and *d* is the bulk density of the sample. The thermal diffusivity was measured by a laser flash technique (Ulvac-Riko, TC-9000h) in the argon atmosphere. The bulk density of the sample was calculated by the Rietveld analysis, and the realistic density was calculated by the principle of the floating bodies of Archimedes. Microscope image and phase composition is analysized by an Electro-Probe Microanalyzer (EPMA) (JXA-8530F, JEOL, Japan).

### Computational details

In order to identify the effect of Sn atoms partially heterovalent substituted by the Ga atoms to the thermoelectric performance of Cu_2_CdGa_x_Sn_1-x_Se_4_, we performed a density functional theory (DFT) study of the density of states (DOS) for our materials. In order to model the Sn substitution by Ga, a 2 × 2 × 1 supercell based on the unit cell was constructed, allowing us to investigate different substitution degrees of Cu_2_CdSn_1-*x*_Ga*_x_*Se_4_, with *x* = 0, 0.125 and 0.25. For *x* = 0.1, a larger 5 × 1 × 1 supercell was designed separately. All calculations are performed using the plane-wave projector-augmented wave method^35^,^36^, as implemented in the Vienna *ab initio* simulation package^37^,^38^. The Perdew-Burke-Ernzerhof (PBE)^39^ form of generalized gradient approximation (GGA) is chosen as the exchange-correlation potential. Structural properties and electronic properties are calculated by the PBE+*U* approach^40^ with spin polarization, with a *U* = 4 eV on Cu 3d and Cd 4d states. To obtain reliable optimized structures, the maximum residual force is less than 0.01 eV/Å and energies are converged to within 5 × 10^−6^ eV per atom, and the k-point mesh is set to 9 × 9 × 9 (2 × 2 × 1 supercell) and 4 × 8 × 6 (5 × 1 × 1 supercell). An energy cut-off of 400 eV was used in all cases.

## Author Contributions

F.P. and F.S.L. designed and conducted the project. B.W. and W.Q.A. conducted the experiments. Y.L. and M.X. performed DFT calculations. The data analyses were performed by F.S.L, J.X.Z., Y.L., F.P. and J.Q.L. This manuscript was written by Y.L., J.X.Z. and F.S.L. All authors reviewed this manuscript.

## Supplementary Material

Supplementary Information Supplementary Information

## Figures and Tables

**Table 1:** The chemical compositions of stannite-type structure Cu_2_CdSn_1–x_Ga_x_Se_4_ phases obstained from EPMA and the calculated chemical formula based on the measurement results

x	Cu	Se	Ga	Sn	Cd	Chemical formula
0	26.23(5)	49.01(5)	0	12.37(5)	12.38(5)	Cu_2_Cd_0.944_Sn_0.943_Se_3.736_
0.025	25.95(8)	49.35(5)	0.05(1)	12.36(2)	12.31(4)	Cu_2_Cd_0.949_Sn_0.952_Ga_0.004_Se_3.80_
0.050	25.90(5)	49.17(6)	0.18(1)	12.02(3)	12.73(4)	Cu_2_Cd_0.983_Sn_0.928_Ga_0.014_Se_3.80_
0.750	25.85(5)	49.38(2)	0.48(1)	11.86(4)	12.43(3)	Cu_2_Cd_0.962_Sn_0.918_Ga_0.037_Se_3.821_
0.100	26.91(5)	48.83(9)	0.80(2)	11.62(9)	11.83(8)	Cu_2_CdSn_0.864_Ga_0.069_Se_3.629_
0.125	24.81(9)	49.62(4)	1.46(6)	10.26(9)	13.85(8)	Cu_1.99_Cd_1.12_Sn_0.837_Ga_0.12_Se_4_

**Table 2:** The Lattice parameters *a*, *c* and *V*, reliable factors, weight fraction of impurity phase CdSe or CuSe obstained by Rietveld refinement, carrier concentration *n*, and carrier mobility *μ* for Cu_2_CdSn_1–x_Ga_x_Se_4_ solid solutions at room temperature. The mark of * refers to CuSe phase

*x*	*a* (Å)	*c* (Å)	*V* (Å^3^)	*R_wp_* (%)	*R_exp_* (%)	*R_Bragg_* (%)	*Gof*	CdSe *wt*.%	*n* (×10^19^)	μ (cm^2^V^−1^s^−1^)
0	5.8292(3)	11.4038(3)	387.50(1)	3.67	1.03	2.78	3.57	0.56	0.41	14.14
0.025	5.8285(3)	11.4051(3)	387.45(1)	3.00	1.03	1.01	2.91	0.78	1.43	27
0.05	5.8280(3)	11.4059(3)	387.42(1)	2.82	1.03	0.96	2.74	0.50	2.15	41.5
0.075	5.8272(3)	11.4055(3)	387.29(1)	2.77	1.02	0.88	2.71	0.30	2.71	53.9
0.1	5.8216(3)	11.4068(3)	386.59(1)	2.72	1.03	1.41	2.65	0.10*	6.95	19.2
0.125	5.8245(3)	11.4125(3)	387.16(1)	3.21	1.04	2.86	3.10	0.41*	7.18	21.6

*refers to CuSe phase.
