# Supplementary material for: Heterovalent Substitution to Enrich Electrical Conductivity in Cu2CdSn1-xGaxSe4 Series for High Thermoelectric Performances
Source: Sci Rep. 2015 Mar 20;5:9365. doi: 10.1038/srep09365 (PMC4366858; doi:10.1038/srep09365)
Supplement: Supplementary Information [file srep09365-s1.pdf]

Supplementary Information

Heterovalent Substitution to Enrich Electrical Conductivity in $Cu_2CdSn_{1-x}Ga_xSe_4$ Series for High Thermoelectric Performances

Bo Wang[1,†], Yu Li[1,†], Jiaxin Zheng[2,†], Ming Xu[2], Fusheng Liu[1,*], Weiqing Ao[1], Junqing Li[1], and Feng Pan[2,*]

[1]College of Materials Science and Engineering, Shenzhen University and Shenzhen Key Laboratory of Special Functional Materials, Shenzhen 518060, People's Republic of China.

[2]School of Advanced Materials, Peking University, Shenzhen Graduate School, Shenzhen 518055, People's Republic of China.

[†]These authors contributed equally to this work.

*Corresponding author: fsliu@szu.edu.cn (F.S. Liu) and panfeng@pkusz.edu.cn (F. Pan)

Table S1. The fractional coordinate of Se atom and the atomic bond-length in $Cu_2CdSn_{1-x}Ga_xSe_4$ structure.

x	$Se_{x,\,y}$	Se_z	Cu-Se (Å)	Cd-Se (Å)	Sn-Se (Å)
0	0.257(1)	0.137(1)	2.43(1)	2.63 (1)	2.54(1)
0.025	0.258(1)	0.137(1)	2.43(1)	2.64(1)	2.54(1)
0.050	0.256 (1)	0.137(1)	2.43(1)	2.62(1)	2.54 (1)
0.075	0.255 (1)	0.136(1)	2.44(1)	2.61(1)	2.54(1)
0.100	0.256 (1)	0.136(1)	2.44(1)	2.62(1)	2.54 (1)
0.125	0.256 (1)	0.135(1)	2.44(1)	2.61(1)	2.53(1)

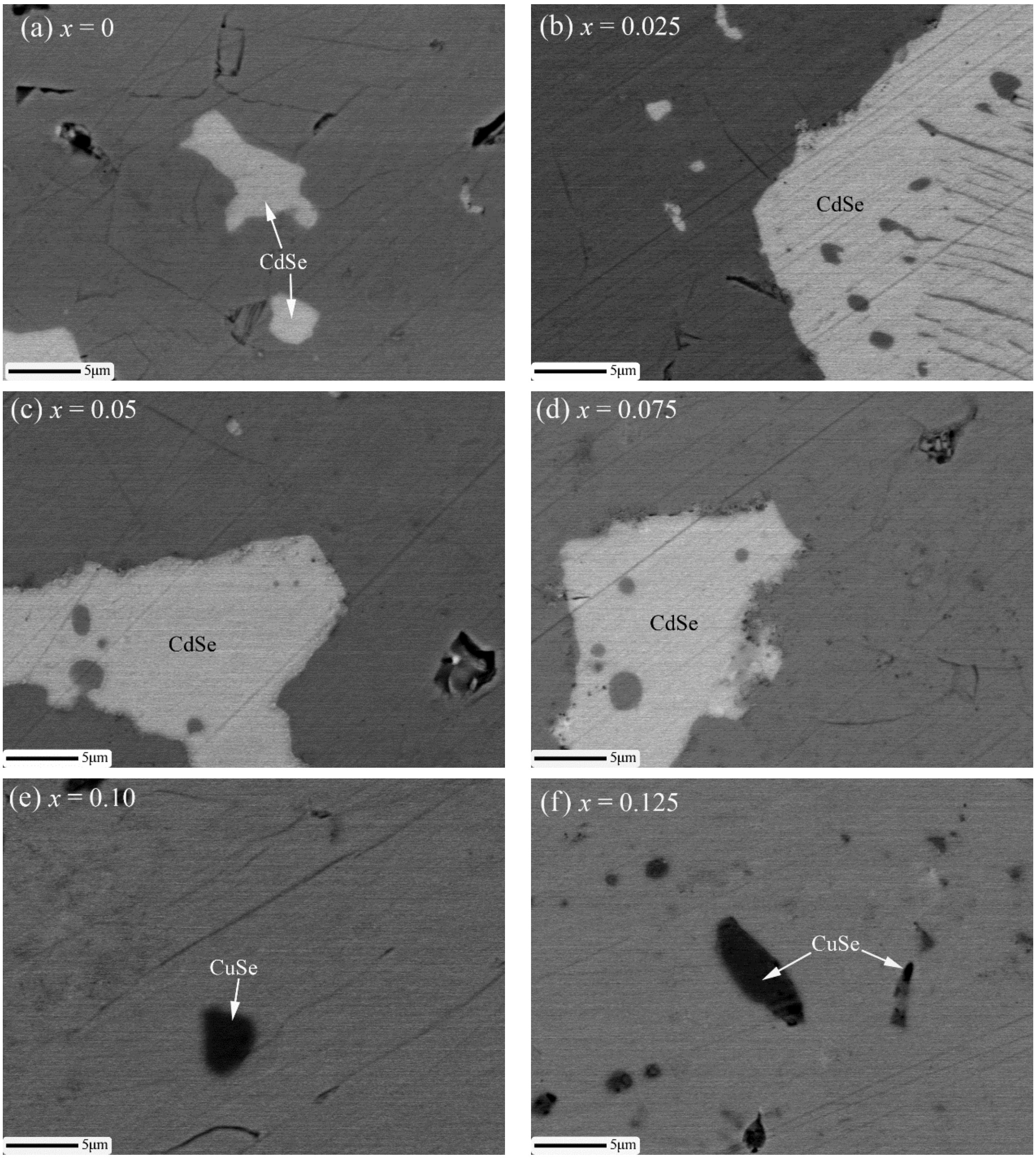

Figure S1. The microscopic images of serials of $Cu_2CdSn_{1-x}Ga_xSe_4$ samples obstained by EPMA to show the second phase in the samples. The black dots in thses images are dusts absorbed on the sample surface.

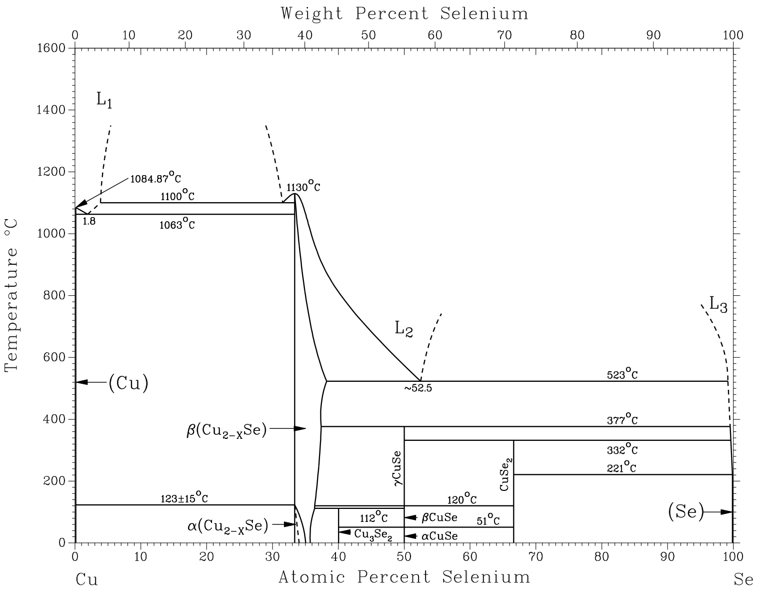

Figure S2. Assessed Cu-Se phase diagram.

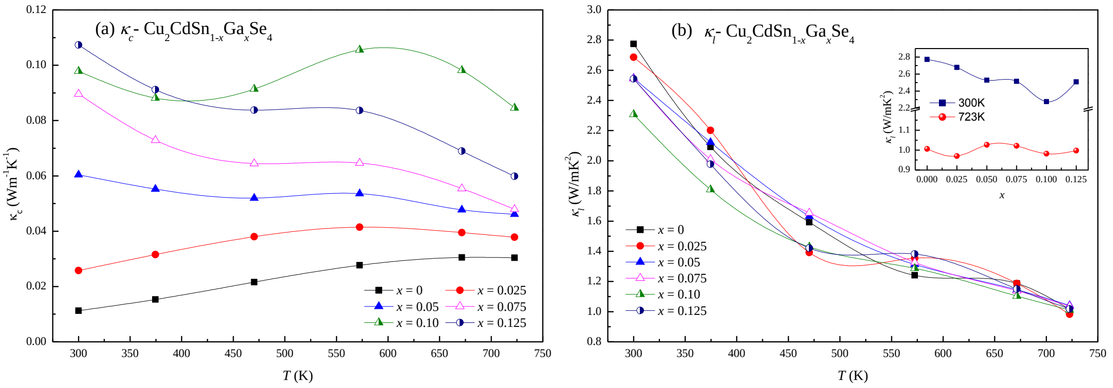

Figure S3. Temperature dependence of thermal transport properties of $Cu_2CdSn_{1-x}Ga_xSe_4$ samples. (a) calculated carrier thermal conductivity(κ_c); (b) lattice thermal conductivity (κ_l), the inset shows the relationship between κ_l and the Ga content x at 300 K and 723 K, respectively.

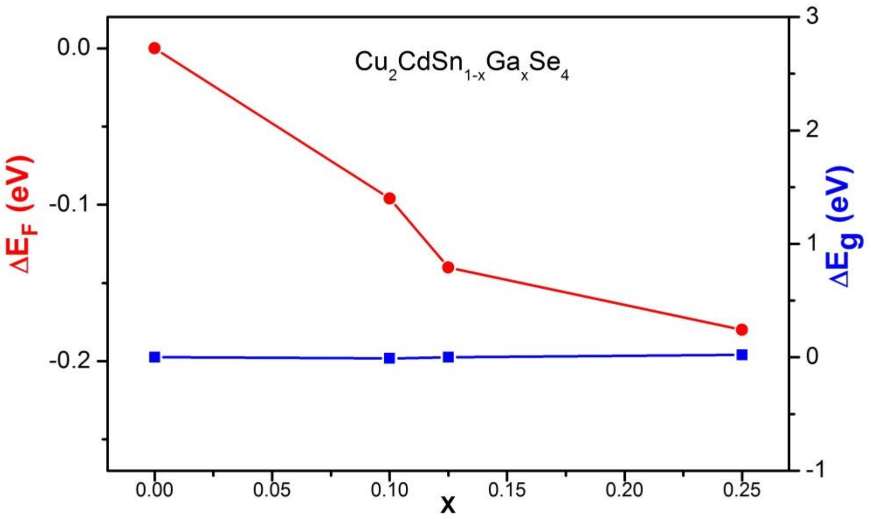

Figure S4. Fermi level shift (red) and band gap variation (blue) as a function of the level of Sn substitution by Ga.
